# Coarctation of the abdominal aorta associated with renovascular hypertension and incapacitating claudication: repair with iliac-birenal bypass and paving and cracking technique

**DOI:** 10.1590/1677-5449.200175

**Published:** 2022-01-17

**Authors:** Mateus Picada Corrêa, Francisco Costa Beber Lemanski, Jaber Nashat Saleh, Rafael Stevan Noel, Renan Camargo Puton, Julio Cesar Bajerski

**Affiliations:** 1 Instituto Vascular de Passo Fundo – INVASC, Passo Fundo, RS, Brasil.; 2 Universidade de Passo Fundo – UPF, Passo Fundo, RS, Brasil.

**Keywords:** abdominal aortic coarctation, iliac-birenal bypass, refractory hypertension, paving and cracking, endovascular

## Abstract

Coarctation of the abdominal aorta is a rare etiology of intermittent claudication and refractory hypertension. Treatment is complex and requires knowledge of several vascular reconstruction techniques. We report a case of aortic coarctation at the level of the renal arteries, describing its treatment and presenting a literature review. Female patient, 65 years old, with refractory hypertension since the age of 35, using five antihypertensive medications at maximum doses. Blood pressure was 260/180mmHg and she had disabling claudication (less than 20 meters). Computed tomography angiography showed a 4mm coarctation in the juxtarenal aorta, with circumferential calcification at the stenosis site, and tortuous infrarenal aorta. Hybrid repair was performed with an iliac-birenal bypass and implantation of an Advanta V12 stent at the stenosis site. The patient’s postoperative course was satisfactory, she was free from claudication, and her blood pressure 60 days after surgery was 140/80mmHg, taking two antihypertensive medications.

## INTRODUCTION

Coarctation of the aorta is a rare congenital disease and when located at the level of the abdominal aorta it constitutes an even rarer variant, accounting for 0.5-2% of all coarctations.^1^^,^^2^ When located at the level of the abdominal aorta, it is a cause of mid aortic syndrome (MAS) and is generally associated with renovascular hypertension and lower limb claudication.

Suspicion is aroused on the basis of patient history and physical examination and diagnosis is confirmed with supplementary examinations such as computed tomography angiography (angio-CT). Several techniques for surgical intervention have been described as treatments for MAS.^2^ In this article, we describe treatment combining iliac-birenal bypass with stenting accomplished using the paving and cracking technique in an adult patient with coarctation of the juxtarenal abdominal aorta.

The Research Ethics Committee approved this study (decision number 5.151.144).

## CASE DESCRIPTION

The patient was a 65-year-old woman who sought care in January 2015 complaining of bilateral intermittent claudication with onset 2 years previously that had progressed to the point at which she was able to walk less than 10 meters. She had had systemic arterial hypertension (SAH) since the age of 35 years, refractory to clinical treatment, and was on the maximum dosages of Metoprolol, Enalapril, Hydrochlorothiazide, Amlodipine, and Losartan, with recurrent hypertensive crises. She had suffered a hemorrhagic stroke 5 years previously, with complete remission of symptoms thereafter.

On physical examination, her blood pressure (BP) was 260/180mmHg and she had complete absence of lower limb pulses bilaterally. Neurological examination detected no sequelae.

**Figure 1:**
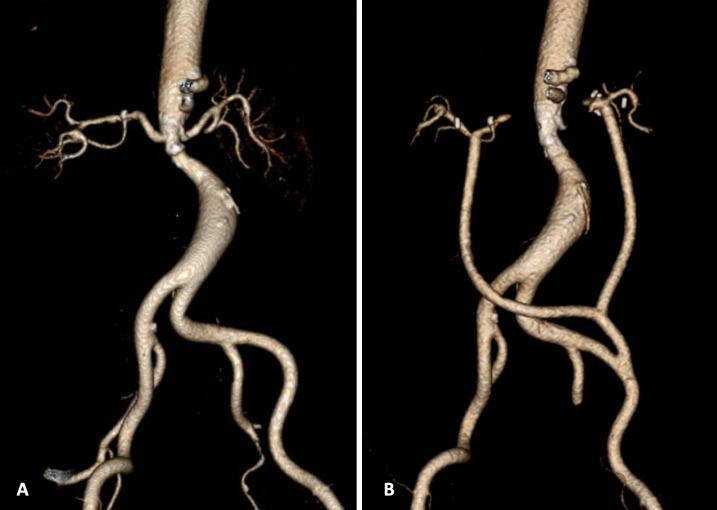
3D reconstruction of computed tomography angiography of the abdominal aorta. (A) Preoperative image showing reduction in the caliber of the aorta at the level of the renal arteries, with parietal calcifications and discrete post-stenotic dilatation; (B) Postoperative control image showing larger diameter at the level of the stenosis, with the Y-bypass to both renal arteries, from the left iliac artery.

**Figure 2:**
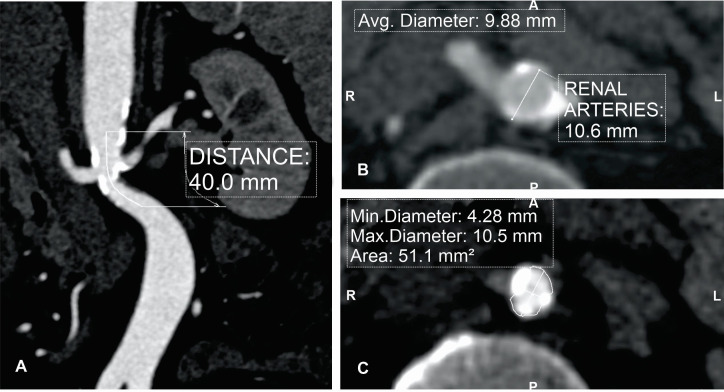
Maximum intensity projection (MIP) reconstruction of the preoperative angiotomography. (A) Coronal slice at the level of the renal arteries showing the parietal calcifications at the level of the renal arteries and the length of stenosis. Axial slices showing; (B) the diameter of the aorta at the level of the renal arteries; and (C) diameter at the point of greatest stenosis.

Abdominal angio-CT revealed coarctation of the aorta at the level of the renal arteries, with a smallest diameter of 4 mm (Figure 1A), circumferential calcification at the stenosis site, and a tortuous infrarenal aorta (Figure 2). In view of the high risk of surgical treatment, she initially opted for clinical treatment but, after claudication worsened, decided to undergo a procedure 3 months later.

**Figure 3:**
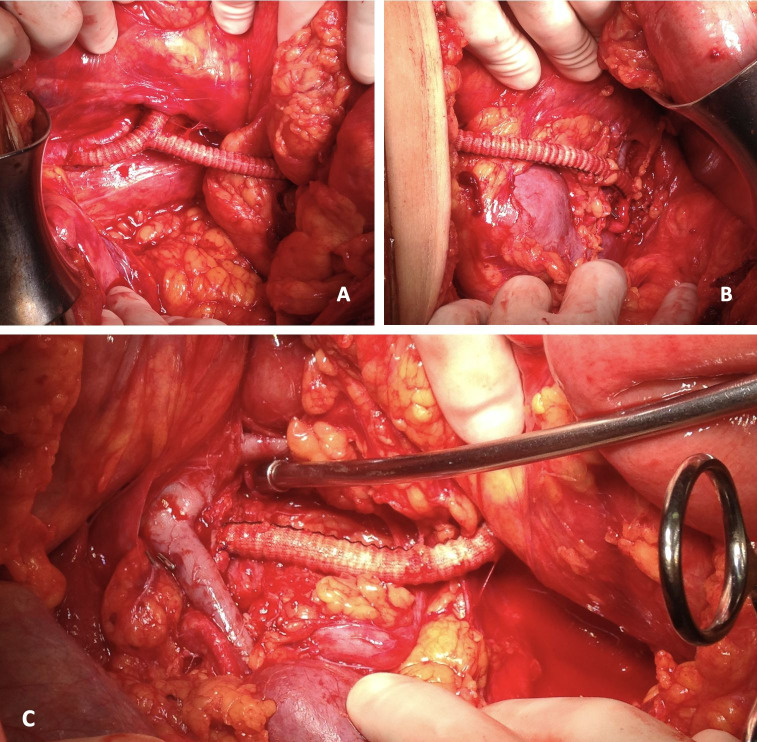
Intraoperative images showing the iliac-prosthesis anastomosis (A – left view), anastomosis of the prosthesis with the left renal artery (B – left view), and anastomosis of the prosthesis with the right renal artery (C – right view).

Using a transverse supraumbilical approach, a bypass was constructed from the left external iliac artery to the left renal artery and then a second bypass was made from the first bypass to the right renal artery using 6 mm Dacron grafts with end-to-side anastomosis, resulting in a Y-shaped birenal bypass, (Figure 3). The prosthesis was assembled on the back table by a second team during the laparotomy. After the bypass had been constructed, angioplasty of the coarctation of the abdominal aorta infrarenal was performed with deployment of a 10 x 38 mm Advanta v12 stent (Getinge AB, Sweden), using a 12 mm x 2 cm Powerflex Pro balloon (Cordis, Santa Clara, EUA) with good angiographic results (Figure 1B). The procedure duration was 5 hours, with bleeding estimated at 400 mL, and transfusion was unnecessary. Venous thromboembolism (VTE) prophylaxis was initiated 24 h after the procedure.

The patient recovered satisfactorily, with no intercurrent conditions during the postoperative period (PO). She was discharged from Intensive care on the third day of PO and discharged from hospital on the seventh. At hospital discharge her BP was 160/140mmHg, she was taking three antihypertensive medications, and she had strong distal pulses.

The patient remained asymptomatic for 1 year and is in her fifth year of follow-up. We repeat angioplasty of the stent with a 12x40 mm balloon annually, on every occasion 30 days after the patient’s pressure begins to increase once more. She has not had claudication again. Her mean BP is 140/80mmHg and she is only taking two antihypertensive medications (Amlodipine and Losartan).

## DISCUSSION

Coarctation of the aorta is a congenital genetic defect that results in narrowing of the aorta. Coarctation can involve any of its segments, but it is most frequently seen at the insertion of the ductus arteriosus, distal of the origin of the left subclavian artery. Coarctation of the aorta is a rare anomaly, accounting for 4 to 6% of all congenital cardiac malformations.^3^ It is generally diagnosed in the first years of life, once the alarm has been raised by SAH, but, depending on the degree of stenosis, it can remain undetected into adulthood.

Coarctation of the aorta at the abdominal level is a rare disease, most often seen among children and adolescents, causing segmental narrowing of the abdominal aorta, and accounting for 2% of all coarctations of the aorta.^4^ It is part of mid aortic syndrome, constituting one of its less common etiologies.^1^^,^^2^^,^^4^^,^^5^ It is believed to be a result of incomplete fusion of the dorsal aortas in the fourth week of gestation. It is a congenital disease of unknown etiology, which may be idiopathic, genetic (Von Recklinghausen’s disease), or acquired (Takayasu’s Arteritis and rubella).^1^^,^^2^

Symptoms are dependent on the location of stenosis, and findings caused by SAH are the most prevalent. The course of juxtarenal coarction involves stenosis of the renal arteries, stimulating release of renin by juxtaglomerular cells of the kidney, causing secondary arterial hypertension. If stenosis is not significant, the malformation may go undetected until adulthood without remarkable symptoms. When present, clinical manifestations are related to severe arterial hypertension, such as headaches, epistaxis, heart failure, or aortic dissection. Clinical history may also include lower limb claudication due to reduced blood flow. Use of angio-CT for diagnosis and preoperative assessment is well-established, providing important information on the arterial anatomy of the region involved.^6^^,^^7^

**Figure 4:**
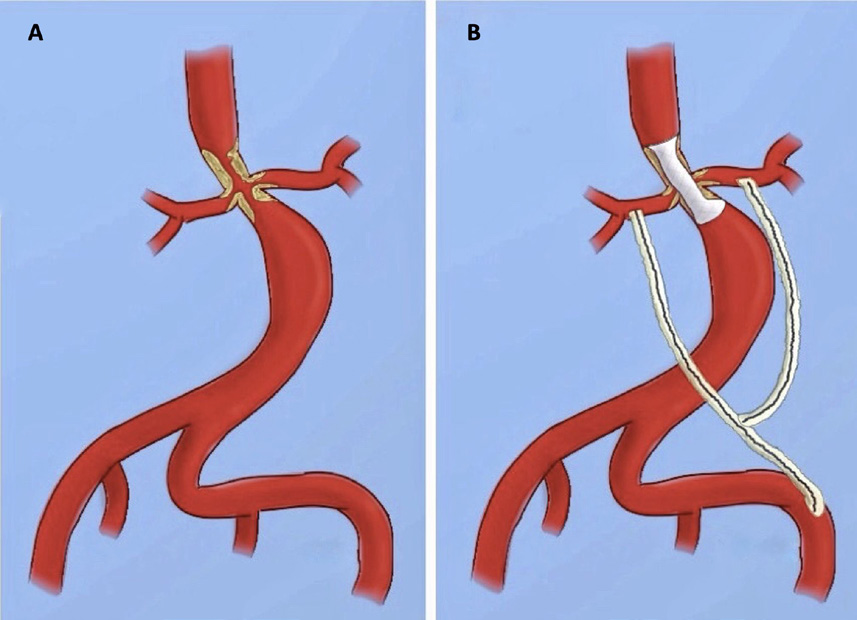
Diagram illustrating the surgical procedure: (A) preoperative and (B) postoperative.

Surgical treatment is indicated for control of SAH refractory to clinical treatment and to alleviate claudication.^2^^,^^7^ The operating technique should be individualized, taking account of the anatomy of the lesion, involvement of visceral arteries and the patient’s cardiovascular risk.^6^ Several techniques can be used for reconstruction of the renal arteries and the aorta: aorto-aortic bypass, patch aortoplasty, endovascular angioplasty, or reconstruction of the renal artery and visceral arteries.^2^^,^^6^^-^8 In the patient described, the procedure chosen was an iliac-birenal bypass for reconstruction of the renal vascularization followed by placement of a stent using “paving and cracking”, to repair the coarctation of the abdominal aorta (Figure 4). There are only 17 cases in the global literature mentioning coarctation of the abdominal aorta in adults over the age of 20 years, since most are diagnosed in childhood, and there are no cases in the Brazilian literature.^6^^,^^9^^-^17

Given the anatomy of this lesion, certain considerations related to planning the procedure should be mentioned. First, in situ reconstruction of the aorta with re-anastomoses of the renal arteries was ruled out in order to avoid suprarenal clamping and simultaneous bilateral renal ischemia, which could increase postoperative morbidity and risk acute renal failure.^18^^,^^19^ Constructing a bilateral iliac-renal bypass with end-to-side anastomosis avoided prolonged renal ischemia. The configuration of the bypass was extrapolated from that recommended by Oderich et al. for retrograde iliac-superior mesenteric reconstruction, since it increases long-term patency by reducing the risk of turbulence.^20^

The choice of a transverse approach rather than a longitudinal incision was suggested by the urology team, with the aim of facilitating access to the renal hila, and the convenience of this approach was confirmed intraoperatively. The wide approach enabled access from the bifurcation of the common iliac artery to the renal arteries, which were our target.

**Figure 5:**
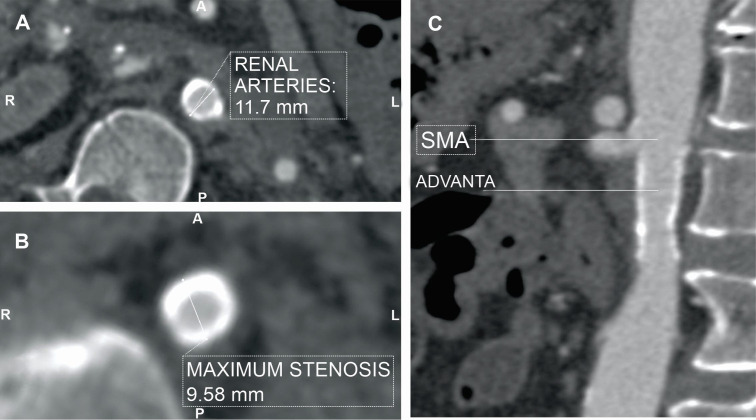
Maximum intensity projection (MIP) reconstruction of the postoperative control angiotomography showing the same slices as in Figure 2; (A) coronal slice at the level of the renal arteries showing the appearance after stenting, and axial slices showing (B) the diameter of the aorta at the level of the renal arteries and (C) the diameter of the aorta at the point of greatest stenosis. SMA = superior mesenteric artery.

The 10x38 v12 stent was chosen because it was the largest diameter covered stent available at the time, offering the option of increasing its diameter to 12 mm with a non-complacent balloon (Figure 5). The decision to use a covered stent reduced the operating time because there was no need to access the ostia of the renal arteries, creating a proximal “endovascular ligature”. Additionally, covered stents are indicated for the “paving and cracking” technique.^21^

This technique was developed to facilitate placement of aortic endoprostheses via severely calcified iliac arteries. After access to the common femoral arteries and angioplasty of the iliac artery, an endoprosthesis or covered stent with diameter compatible with the artery is deployed, with the aim of realigning the arterial lumen (paving). Next, the artery is over-dilated with a 10 mm diameter balloon along the entire stent, fragmenting the plaque (cracking). The stent material prevents distal embolization or bleeding. Since the iliac artery has been lined and dilated, the aortic endoprosthesis can then be easily inserted and deployed in the standard manner.^21^ In this case, we performed over-dilation with the 12 mm balloon, and the covered stent prevented bleeding, if there had been any rupture of the artery wall.

## CONCLUSIONS

Coarctation of the abdominal aorta is a rare etiology of symmetrical lower limb claudication or refractory hypertension; however, when these symptoms are seen together, this diagnosis should always be considered. Treatment is complex, demanding knowledge of several different vascular reconstruction techniques for success, and the objective is to reverse SAH and claudication.
