# Supplementary material for: Coarctation of the abdominal aorta associated with renovascular hypertension and incapacitating claudication: repair with iliac-birenal bypass and paving and cracking technique
Source: J Vasc Bras. 2022 Jan 17;21:e20200175. doi: 10.1590/1677-5449.200175 (PMC8786083; doi:10.1590/1677-5449.200175)
Supplement: Translation

# Translation: Coarctação de aorta abdominal justarrenal associada à hipertensão renovascular e claudicação incapacitante: tratamento utilizando ponte ilíaco-birrenal e técnica “*paving and cracking*”

**Authors:** Mateus Picada Corrêa, Francisco Costa Beber Lemanski, Jaber Nashat Saleh, Rafael Stevan Noel, Renan Camargo Puton, Julio Cesar Bajerski

**Affiliations:** 1 Instituto Vascular de Passo Fundo – INVASC, Passo Fundo, RS, Brasil.; 2 Universidade de Passo Fundo – UPF, Passo Fundo, RS, Brasil.

**Keywords:** coarctação de aorta abdominal, ponte ilíaco-birrenal, hipertensão refratária, paving and cracking, endovascular

## Abstract

Coarctação da aorta abdominal é uma causa rara de claudicação de membros inferiores e hipertensão refratária. O tratamento é complexo e exige conhecimento de diversas técnicas de reconstrução vascular. Apresentamos um caso de coarctação ao nível das artérias renais, seu tratamento e revisão da literatura. Paciente feminina, 65 anos, com hipertensão refratária desde os 35 anos, utilizando cinco medicações anti-hipertensivas em dose máxima. Pressão arterial média de 260/180mmHg e claudicação incapacitante (menos de 20 metros) bilateral. Angiotomografia computadorizada demonstrou coarctação de aorta justarrenal de 4 mm de maior diâmetro, calcificação circunferencial no local da estenose e tortuosidade da aorta infrarrenal. Foi submetida a tratamento híbrido, com ponte ilíaco-birrenal e implante de stent Advanta V12 no local da estenose. A paciente evoluiu satisfatoriamente e, 60 dias depois da cirurgia, apresentava-se com uma pressão arterial de 140/80mmHg, em uso de apenas duas medicações anti-hipertensivas e sem claudicação.

## INTRODUÇÃO

Coarctação da aorta é uma doença congênita rara, e a sua localização ao nível da aorta abdominal é uma variante ainda mais incomum da doença, representando 0,5-2% de todas as coarctações^1^^,^^2^. Quando está localizada ao nível da aorta abdominal, é uma causa de síndrome da aorta média (SAM) e geralmente está acompanhada de hipertensão renovascular e claudicação de membros inferiores.

A suspeita é feita com base na anamnese e no exame físico. A confirmação diagnóstica é feita com exames complementares, como a angiotomografia computadorizada (angio-TC). Diversas técnicas de intervenção cirúrgica foram descritas para o tratamento da SAM^2^. Neste artigo, relatamos o tratamento combinado de *bypass* ilíaco-birrenal com implante de stent por meio da técnica *paving and cracking* em uma paciente adulta com coarctação de aorta abdominal justarrenal.

O protocolo foi aprovado pelo Comitê de Ética de nossa instituição (parecer número 5.151.144).

## DESCRIÇÃO DO CASO

Paciente do sexo feminino, 65 anos, procura atendimento em janeiro de 2015 com queixa de claudicação intermitente bilateral há 2 anos, agora, para menos de 10 metros. Apresentava hipertensão arterial sistêmica (HAS) desde os 35 anos, refratária ao tratamento clínico, em uso de dose máxima de Metoprolol, Enalapril, Hidroclorotiazida, Anlodipino e Losartana, com crises hipertensivas de repetição. Refere história de acidente vascular encefálico hemorrágico há cinco anos, com remissão completa dos sintomas.

Ao exame físico, a pressão arterial (PA) era de 260/180mmHg e demonstrava ausência total de pulsos bilateral nos membros inferiores. O exame neurológico revelou não ter sequelas.

A angio-TC de abdome demonstrou coarctação de aorta ao nível das artérias renais, medindo 4 mm de menor diâmetro (Figura 1A), com calcificação circunferencial no local da estenose e tortuosidade da aorta infrarrenal (Figura 2). Em virtude do tratamento cirúrgico de alto risco, a paciente optou por uma tentativa de tratamento clínico momentaneamente e, por piora da claudicação, optou por realizar procedimento três meses depois.

**Figura 1:** Reconstrução 3D de angiotomografia computadorizada de aorta abdominal. (A) Exame pré-operatório demonstrando redução do calibre da aorta ao nível das artérias renais, com calcificações parietais e discreta dilatação pós-estenótica; (B) Exame de controle pós-operatório demonstrando o aumento do diâmetro ao nível da estenose, com a ponte em Y para ambas as artérias renais, de origem da artéria ilíaca esquerda.

**Figura 2:** (A) Reconstrução em *maximum intensity projection* (MIP) da angiotomografia pré-operatória demonstrando um corte coronal ao nível das artérias renais, evidenciando as calcificações parietais ao nível das renais e o comprimento da estenose; (B) Cortes axiais demonstrando o diâmetro aórtico ao nível das artérias renais; (C) Diâmetro no ponto de maior estenose.

Através de um acesso transverso supraumbilical, foi realizada uma ponte da artéria ilíaca externa esquerda para a artéria renal esquerda e, desta, uma ponte em Y para a artéria renal direita, utilizando Dacron 6 mm com anastomose término-lateral (Figura 3). A prótese foi montada em bancada por uma segunda equipe durante a laparotomia. Após o término do *bypass*, foi realizada angioplastia da área da coarctação da aorta abdominal infrarrenal com implante de stent Advanta v12 10 x 38 mm (Getinge AB, Suécia) e balão Powerflex Pro 12 mm x 2 cm (Cordis, Santa Clara, EUA), tendo bom resultado angiográfico (Figura 1B). O procedimento teve duração de 5 horas, com sangramento estimado de 400 mL e sem necessidade de transfusão sanguínea. Foi iniciada profilaxia de tromboembolismo venoso (TEV) 24 h após o procedimento.

**Figura 3:** Imagens transoperatórias demonstrando a anastomose ilíaco-prótese (A – visão à esquerda), a anastomose da prótese com a artéria renal esquerda (B – visão à esquerda) e a anastomose da prótese com a artéria renal direita (C – visão à direita).

A paciente evoluiu satisfatoriamente, sem intercorrências no pós-operatório (PO), recebendo alta do Centro de Terapia Intensiva (CTI) no terceiro dia de PO e alta hospitalar no sétimo. No momento da última alta, apresentava PA de 160/140mmHg, estava em uso de três medicações anti-hipertensivas e possuía pulsos distais amplos.

A paciente permaneceu assintomática por um ano e está no quinto ano de acompanhamento. Anualmente, realizamos angioplastia do stent com balão 12x40 mm, sempre 30 dias depois da pressão da paciente voltar a subir. Não houve mais claudicação. A PA média da paciente é de 140/80mmHg com uso de apenas dois medicamentos anti-hipertensivos (Anlodipino e Losartana).

## DISCUSSÃO

A coarctação de aorta é um defeito genético congênito que resulta no estreitamento da aorta. A coarctação pode afetar qualquer dos seus segmentos, porém é mais frequentemente encontrada na inserção do canal arterial, distal à emergência da artéria subclávia esquerda. Sendo uma anomalia infrequente, a coarctação de aorta corresponde de 4 a 6% de todas malformações cardíacas congênitas^3^. Seu diagnóstico geralmente é feito nos primeiros anos de vida, sendo a HAS o sinal alarmante, porém a alteração pode passar despercebida até a idade adulta, dependendo do grau da estenose.

A coarctação da aorta ao nível abdominal é uma doença rara, mais vista em crianças e adolescentes, que gera um estreitamento segmentar da artéria aorta abdominal, representando 2% de todas coarctações de aorta^4^. Faz parte da síndrome da aorta média, sendo uma de suas etiologias menos comuns^1^^,^^2^^,^^4^^,^^5^. Acredita-se ser decorrente da fusão incompleta das aortas dorsais na quarta semana de gestação. É uma doença congênita de etiologia desconhecida, podendo ter causa idiopática, genética (doença de Von Recklinghausen) ou adquirida (arterite de Takayasu e rubéola)^1^^,^^2^.

Os sintomas dependem da topografia da estenose, sendo os achados decorrentes da HAS os mais prevalentes. Coarctação justarrenal cursa com estenose das artérias renais, gerando estímulo à liberação da renina das células justaglomerulares do rim, causando hipertensão arterial secundária. Se a estenose não for significativa, a malformação pode passar despercebida até a idade adulta sem sintomas expressivos. Quando presentes, as manifestações clínicas estão relacionadas à hipertensão arterial grave, como cefaleia, epistaxe, insuficiência cardíaca ou dissecção aórtica. Além disso, na história clínica, pode haver claudicação dos membros inferiores devido à redução do fluxo sanguíneo. O uso da angio-TC para diagnóstico e avaliação pré-operatória está bem-estabelecido, fornecendo informações importantes acerca da anatomia arterial da região acometida^6^^,^^7^.

O tratamento cirúrgico é indicado para o controle da HAS refratária ao tratamento clínico e para melhora da claudicação^2^^,^^7^. A técnica operatória deve ser individualizada, levando em consideração a anatomia da lesão, o envolvimento de artérias viscerais e o risco cardiovascular do paciente^6^. Diversas técnicas podem ser utilizadas para a reconstrução renovascular e da aorta: *bypass* aorto-aórtico, aortoplastia com retalho, angioplastia endovascular, reconstrução de artéria renal e de artérias viscerais^2^^,^^6^^-^8. Na paciente relatada, foi optado pelo *bypass* ilíaco-birrenal para reconstrução da vascularização renal e, em seguida, a implatação do stent por “*paving and cracking*” para reparo da coartação da aorta abdominal (Figura 4). Existem apenas 17 casos mencionados na literatura mundial sobre a ocorrência de coarctação de aorta abdominal em adultos com idade superior a 20 anos, visto que a maior parte dos diagnósticos ocorrem na infância, e nenhum na literatura brasileira^6^^,^^9^^-^17.

**Figura 4:** Representação esquemática do procedimento cirúrgico no pré- (A) e pós-operatório (B).

Dada a anatomia da lesão, algumas considerações a respeito do planejamento do procedimento devem ser destacadas. Primeiro, não foi optada pela reconstrução *in situ* da aorta com reanastomose das artérias renais para se evitar o clampeamento suprarrenal e a isquemia renal bilateral simultânea, o que poderia aumentar a morbidade pós-operatória com risco de insuficiência renal aguda^18^^,^^19^. Realizando uma ponte ilíaco-renal bilateral com anastomose término-lateral, não tivemos isquemia renal prolongada. A conformação do *bypass* foi extrapolada da recomendada por Oderich et al. para as reconstruções retrógradas ilíaco-mesentérica superior, que aumentam a perviedade em longo prazo por reduzir o risco de turbilhonamento^20^.

A opção de realizar um acesso transverso em vez de longitudinal foi sugerida pela equipe de Urologia, a fim de facilitar o acesso aos hilos renais, o que se confirmou conveniente no transoperatório. Com o amplo acesso, foi possível acessar desde a bifurcação da artéria ilíaca comum até as artérias renais, nosso alvo.

O stent v12 10x38 foi escolhido por ser, na ocasião, o *stent* revestido de maior diâmetro disponível, tendo a opção de aumentar o diâmetro para 12 mm com um balão não complacente (Figura 5). A decisão de se utilizar stent revestido reduziu o tempo cirúrgico por não haver necessidade de acesso aos óstios das artérias renais, criando uma “ligadura endovascular” proximal. Além disto, *stents* revestidos são indicados na técnica de “*paving and cracking*”^21^.

**Figura 5:** Reconstrução em *maximum intensity projection* (MIP) da angiotomografia pós-operatória de controle demonstrando os mesmos cortes da Figura 2: um corte coronal ao nível das artérias renais, evidenciando o aspecto do implante do stent (A), e cortes axiais demonstrando o diâmetro aórtico ao nível das artérias renais (B) e o diâmetro no ponto de maior estenose (C). AMS = artéria mesentérica superior.

Essa técnica foi desenvolvida para facilitar o implante de endopróteses aórticas através de artérias ilíacas severamente calcificadas. Após o acesso às artérias femorais comuns e a angioplastia da artéria ilíaca, é realizado o implante de uma endoprótese ou stent revestido de diâmetro compatível com a artéria, no intuito de realinhar o lúmen arterial (*paving*). Sequencialmente, realiza-se uma sobredilatação da artéria com balão de diâmetro de 10 mm em toda a área do stent, fragmentando a placa (*cracking*). O tecido do stent previne embolização distal ou sangramento. Uma vez que a artéria ilíaca tenha sido revestida e dilatada, a endoprótese aórtica pode ser inserida facilmente e implantada de maneira padrão^21^. No caso em questão, realizamos uma sobredilatação com balão 12 mm, e o *stent* revestido impediu sangramento, caso tenha ocorrido eventual ruptura da parede arterial.

## CONCLUSÃO

A coarctação da aorta abdominal é uma etiologia rara de claudicação simétrica de membros inferiores ou hipertensão refratária; todavia, na associação desses sintomas, o diagnóstico deve sempre ser considerado. O tratamento é complexo e exige conhecimento de diversas técnicas de reconstrução vascular para o seu sucesso, tendo como objetivo reverter a HAS e a claudicação do paciente.
